# Supplementary material for: Biallelic Variants in ENDOG Associated with Mitochondrial Myopathy and Multiple mtDNA Deletions
Source: Cells. 2022 Mar 12;11(6):974. doi: 10.3390/cells11060974 (PMC8946636; doi:10.3390/cells11060974)
Supplement: Supplementary file 1 [file cells-11-00974-s001.zip › cells-1615424-supplementary.pdf]

Supplemental Table S1: Biochemical analysis of OXPHOS complexes

	CI/CS	CII/CS	CI+III/CS	CII+III/CS	CIV/CS	CS*
**Patient**	2.8 (25%)	17.0	9.6 (26%)	4.1 (34%)	7.0 (15%)	239.0 (174%)
**Controls**	11.3±3.3	17.6±2.8	36.6±6.8	12.0±2.1	47.6±6.0	137.3±15.0

CI, CII, CIII, CIV: complexes I, II, III, IV of the Oxidative Phosphorylation system. CS: citrate synthase. *value in nmol/min mg.

Supplemental Table S2: Additional candidate variants from NGS analyses

Gene	cDNA	Refseq	Protein	ACMG	Associated disease	Inheritance
C1QBP **(MIM*601269)**	c.1A>G	NM_001212.3	p.(Met1?)	VUS	MIM#617713	AR
MRPS34 **(MIM*611994)**	c.94C>T	NM_023936.1	p.Gln32*	Pathogenic	MIM#617664	AR

ACMG: criteria according to the American college of Medical Genetics; VUS: variant of uncertain significance; AR: autosomal recessive.

Supplemental Figure S1: Analysis of the mtDNA by Next Generation Sequencing

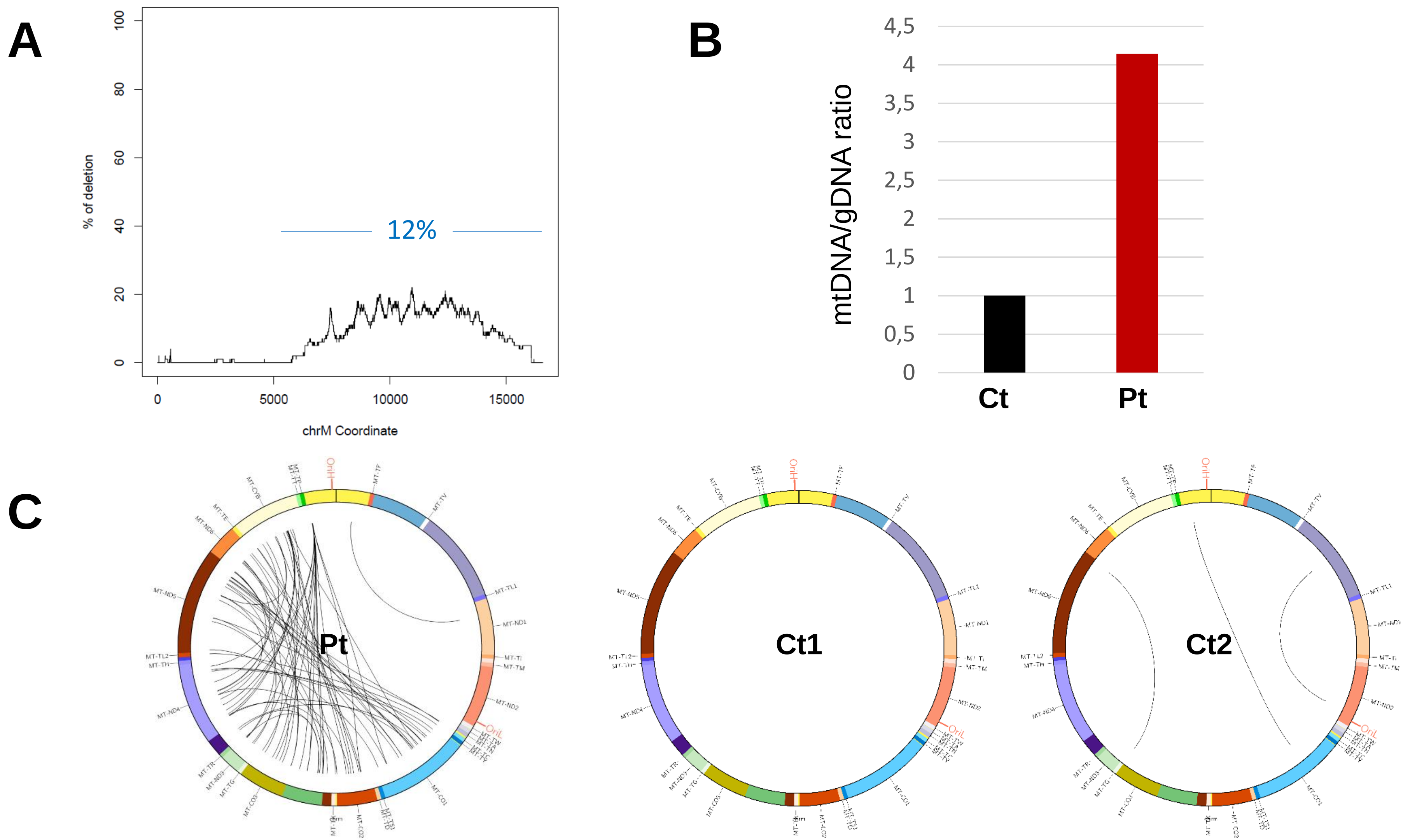

Evaluations of mtDNA in patient muscle DNA: NGS reads quantification showing multiple mtDNA deletions, with mean heteroplasmy of 12% in the affected region (panel **A**); mtDNA content normalized to nuclear DNA (mtDNA/nDNA) in Patient's (Pt) and control (Ct) samples (panel **B**); visualization of the mtDNA deletions by Circos software in Patient (Pt) and age-matched controls (Ct) (panel **C**).

Supplemental Figure S2: Structural analysis of residues affected by the missense variants

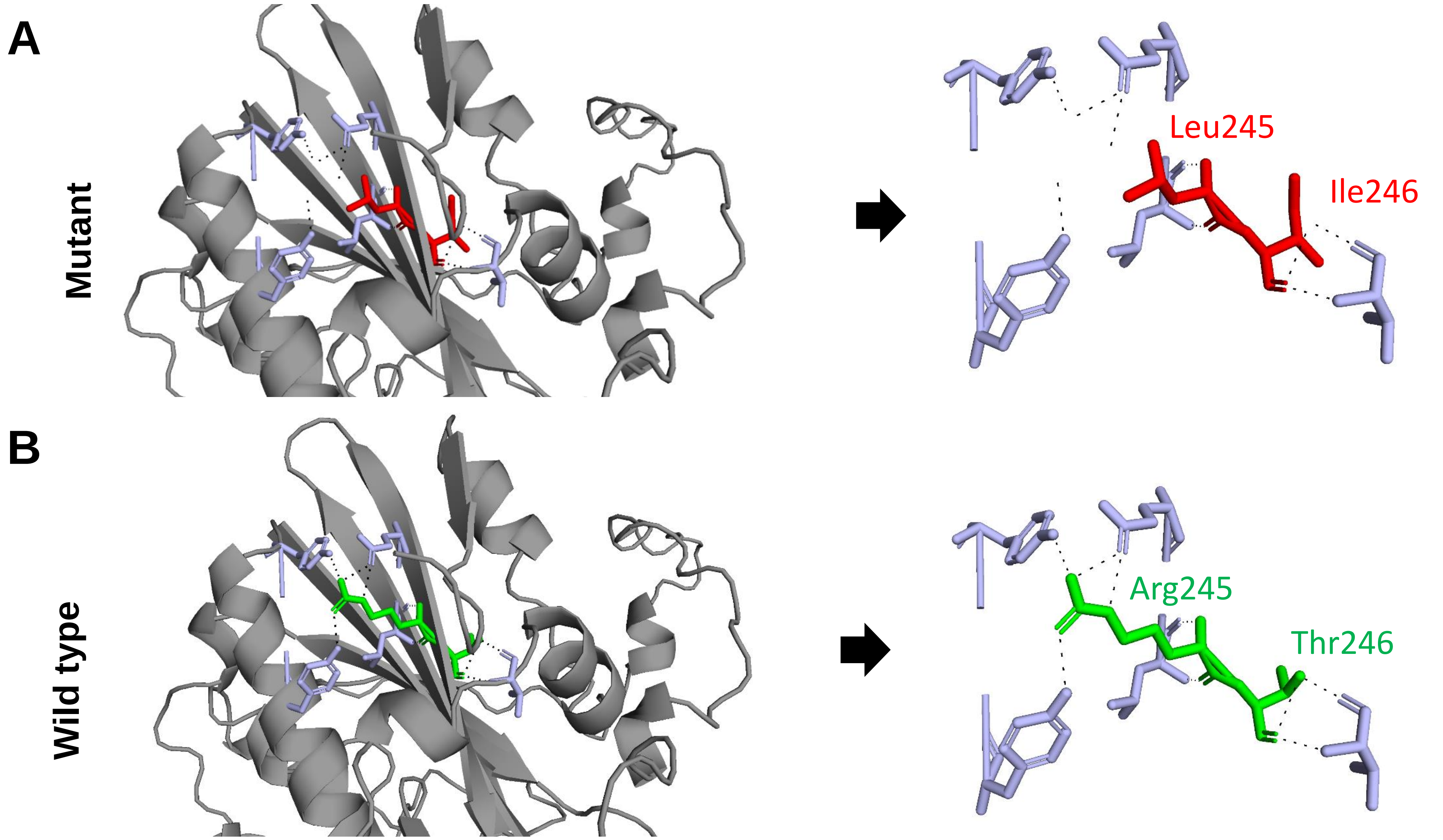

Mapping of the mutant sequence harboring the amino acid substitution p.Arg245Leu-p.Thr246Ile (panels **A**), and the wild type sequence (panels **B**) on ENDOG structure (AF-Q14249-F1). The dotted lines indicate hydrogen bonds. Amino acid numbering is made according to NP_004426.2.

Supplemental Figure S3: Immunofluorescence studies in fibroblasts

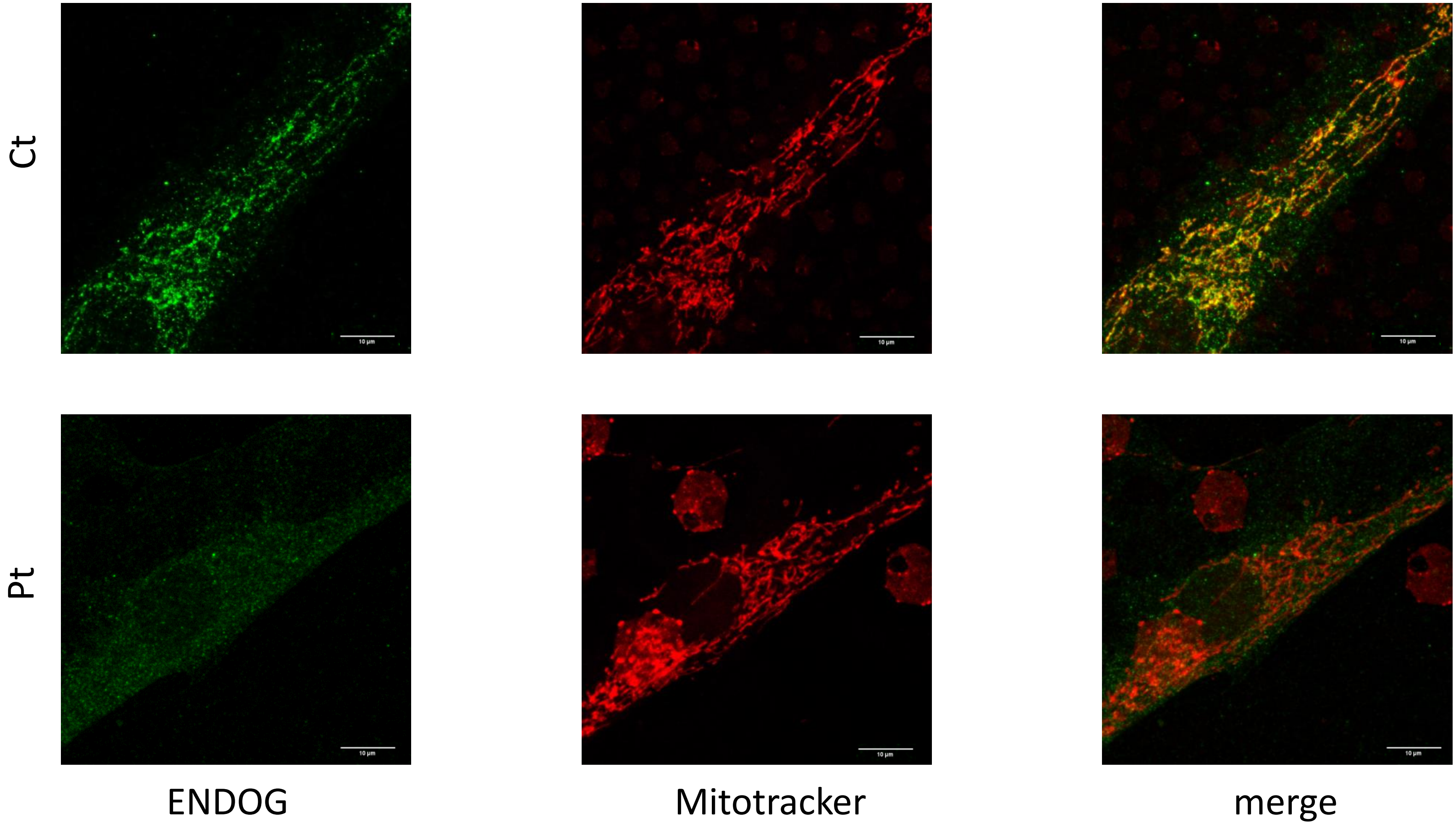

Staining obtained with the anti-ENDOG antibody (green) and the mitochondrial marker Mitotracker Red (red) in fibroblasts from the patient (Pt) and a control (Ct). The same parameters for imaging acquisition were used for Pt and Ct. The merged signals are reported in the right panels. Scale bar: 10 µm.

Supplemental Figure S4: Western blot analysis of C1QBP

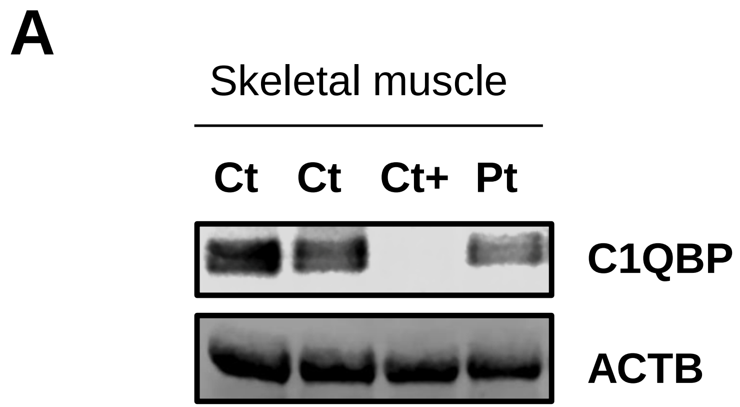

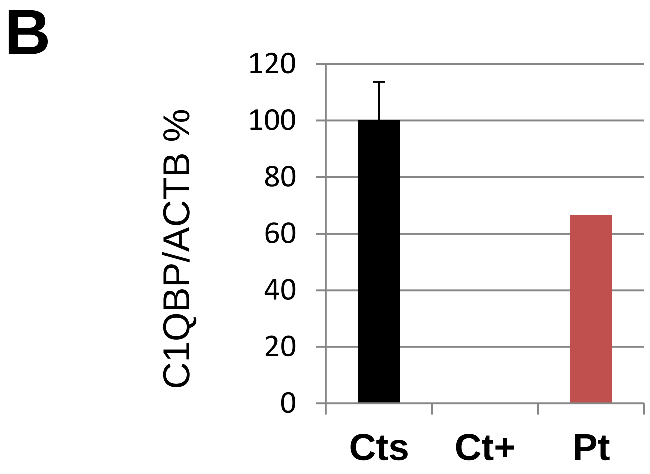

Western blot analysis of C1QBP in skeletal-muscle lysates from control individuals (Ct), a patient harboring biallelic *C1QBP* mutations (Ct+) and the Patient described in the manuscript (Pt). Cytosolic b-actin (ACTB) were used as loading control (panel **A**). Graph reporting protein level normalized to ACTB in percentage, being 100% the mean value of Controls (panel **B**).

Supplemental Figure S5: *ENDOG* transcript analysis in patient's fibroblasts

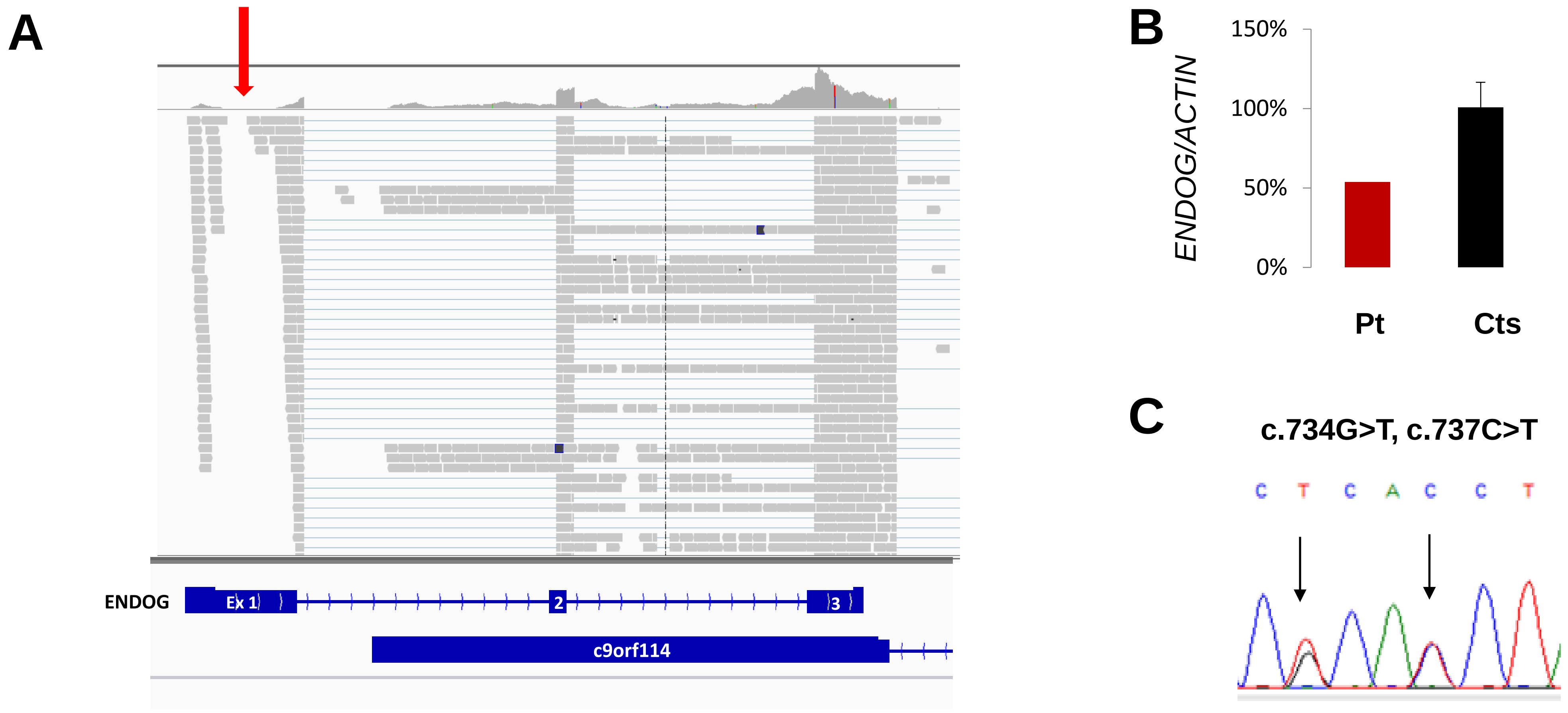

Snapshot from IGV software of the region containing *ENDOG* and C9orf114 transcripts, obtained by RNA sequencing in patient's fibroblasts. The red arrow indicates the position of the c.61delG within exon 1, not captured in the analysis (panel **A**). Graph reporting *ENDOG* transcript level normalized to *ACTIN,* being 100% the mean value of Controls (panel **B**). Electropherograms of the region containing the *ENDOG* missense variants in complementary DNA, retro-transcribed from patient's fibroblasts RNA.
